# Intervention for a Digital, Cognitive, Multi-Domain Alzheimer Risk Velocity Study: Protocol for a Randomized Controlled Trial

**DOI:** 10.2196/31841

**Published:** 2022-02-04

**Authors:** Michelle Gray, Erica N Madero, Joshua L Gills, Sally Paulson, Megan D Jones, Anthony Campitelli, Jennifer Myers, Nicholas T Bott, Jordan M Glenn

**Affiliations:** 1 Exercise Science Research Center Department of Health, Human Performance, and Recreation University of Arkansas Fayetteville, AR United States; 2 Neurotrack Technologies, Inc Redwood City, CA United States

**Keywords:** health coaching, Alzheimer risk, digital health, mobile phone

## Abstract

**Background:**

In the United States, more than 6 million adults live with Alzheimer disease (AD) that affects 1 out of every 3 older adults. Although there is no cure for AD currently, lifestyle-based interventions aimed at slowing the rate of cognitive decline or delaying the onset of AD have shown promising results. However, most studies primarily focus on older adults (>55 years) and use in-person interventions.

**Objective:**

The aim of this study is to determine the effects of a 2-year digital lifestyle intervention on AD risk among at-risk middle-aged and older adults (45-75 years) compared with a health education control.

**Methods:**

The lifestyle intervention consists of a digitally delivered, personalized health coaching program that directly targets the modifiable risk factors for AD. The primary outcome measure is AD risk as determined by the Australian National University-Alzheimer Disease Risk Index; secondary outcome measures are functional fitness, blood biomarkers (inflammation, glucose, cholesterol, and triglycerides), and cognitive function (Repeatable Battery for the Assessment of Neuropsychological Status and Neurotrack Cognitive Battery). Screening commenced in January 2021 and was completed in June 2021.

**Results:**

Baseline characteristics indicate no difference between the intervention and control groups for AD risk (mean −1.68, SD 7.31; *P*=.90).

**Conclusions:**

The intervention in the Digital, Cognitive, Multi-domain Alzheimer Risk Velocity is uniquely designed to reduce the risk of AD through a web-based health coaching experience that addresses the modifiable lifestyle-based risk factors.

**Trial Registration:**

ClinicalTrials.gov NCT04559789; https://clinicaltrials.gov/show/NCT04559789

**International Registered Report Identifier (IRRID):**

DERR1-10.2196/31841

## Introduction

### Background

Alzheimer disease (AD) is a chronic neurodegenerative disorder characterized by the buildup of neurofibrillary tangles and plaques in the brain and a steady decline in memory and executive function. These cognitive changes eventually lead to physical dependence [1] and ultimately death. Currently, AD is the sixth leading cause of death in the United States, climbing from the 12th position in less than 3 decades [2]; mortality rates of other chronic illnesses such as cardiovascular disease and stroke have continued to decrease during this same time frame [3]. Although AD was first identified more than 100 years ago, the advances in disease detection, prevention, and treatment have not been as successful as those in other chronic illnesses. Many cases go undetected for up to 20 years, owing to a long prodromal period, fear of diagnosis, and inability to access proper testing [4].

In addition to being a significant contributor to deaths among US adults, AD is a major burden to the health care system. AD accounts for an estimated US $355 billion in annual direct and indirect health care costs [5]. These costs have been rising dramatically, increasing by 38% in the past year alone [2,5]. As the number of older adults is expected to increase by more than 53% within the next 40 years [6], the number of older adults living with AD will also continue to grow and increase the burden on the health care system, as well as individuals with the disease and their caretakers. So far, pharmacological interventions have been ineffective in generating long-term and sustained improvements in cognitive function [7]. The combination of these circumstances has created a critical need for the development and implementation of effective preventative interventions.

AD is a multifactorial disease that involves several mechanisms that contribute to its development and progression. Nonmodifiable risk factors include advanced age, female sex, and presence of the apolipoprotein E4 (APOE4) allele. Such nonmodifiable risk factors are important to consider when assessing an individual’s risk for developing AD, but nothing can be done to address these factors. However, some modifiable risk factors (eg, lipids, glucose, inflammatory factors, and multiple lifestyle behaviors) have been identified as possible contributors to the disease [8,9]. These risk factors represent important targets for interventions to reduce the risk for developing dementia.

Many modifiable risk factors for vascular disease are known to concurrently contribute to the development of AD [10]. These risk factors include overweight and obesity, physical inactivity, stress, chronic inflammation, dietary habits, sleep, and blood glucose and lipid levels [11]. Additional risk factors for AD include low cognitive activity and low social engagement [11].

### Intervention Strategies

#### Single-Domain Lifestyle Interventions

The average age of AD diagnosis is 75.5 (SD 9.7) years [12], but current research suggest that subtle changes in cognitive function can begin 20 years before diagnosis [4,5]. This early stage of the disease represents an important time during which interventions targeted at the modifiable risk factors could be the most effective.

Several studies have investigated single-domain lifestyle interventions to lower the risk of dementia and AD [7,9,13]. Three popular interventions include cognitive training [7], dietary changes [9,14], or physical activity [13]. Evidence suggests that strict adoption of a healthy diet significantly reduces AD risk [9,14]; however, there is less effect if adherence is not strictly followed. Computerized cognitive training has gained popularity, and research supports a moderate improvement in cognition among healthy adults after training [7]. In addition, aerobic exercise is a possible intervention for cognitive decline among healthy adults. Researchers found that an aerobic exercise intervention was not better than the control for any cognitive domain; however, this review only selected adults without cognitive decline and did not address the individual risk of cognitive decline or dementia [13]. Although single-domain interventions are effective, combining these interventions may provide an even greater benefit.

#### Multi-Domain Lifestyle Interventions

Owing to the limited efficacy of the single-domain interventions, researchers have begun to investigate the use of multi-domain lifestyle interventions that target various factors associated with cognitive decline [15]. One of the largest trials is the Finnish Geriatric Intervention Study to Prevent Cognitive Impairment and Disability trial [16]. This 2-year longitudinal intervention targeted older adults with increased risk of developing dementia, as determined by the Cardiovascular Risk Factors, and Aging, and Dementia risk score [10,15]. The lifestyle intervention in the study addressed nutrition, exercise, cognitive training, and vascular risk monitoring. At the end of the 2-year intervention, participants in the intervention group showed 25% improvement in cognitive assessment scores compared with the control participants, with the greatest improvements occurring in processing speed and executive function [10,15].

Although these results are promising, not all multi-domain interventions have resulted in cognitive improvements. For instance, prevention of dementia by intensive vascular care [17] and Multi-domain Alzheimer Preventative Trial [18] did not positively affect cognitive decline. Thus, further research is needed to determine the optimal elements to include in a multi-domain lifestyle intervention and the best population to target to maximize the benefits. As such, the Digital, Cognitive, Multi-domain Alzheimer Risk Velocity (DCMARVel) trial will assess the impact of a digital lifestyle intervention targeting the modifiable risk factors for AD in middle-aged to older adults compared with a health education (HE) control. The personalized intervention involves health coaching (HC) to address the lifestyle areas known to be linked to AD risk, such as diet, exercise, sleep, stress, cognitive training, and social interaction.

### Purpose

Of the studies on multi-domain lifestyle interventions for reducing AD risk, few have tested middle-aged individuals and few have implemented digital interventions. Using the intervention program earlier in the disease course can maximize its benefits, and using digital technologies rather than relying on face-to-face implementation can greatly increase the reach and scalability of these programs. Therefore, the purpose of the DCMARVel trial is to determine the effects of a 2-year digital, multi-domain lifestyle intervention on AD risk among at-risk middle-aged to older adults (45-75 years). Moreover, the digital and personalized nature of the intervention provides the opportunity to explore the variables that may contribute to the accessibility and subsequent adherence to an AD risk reduction intervention. This paper outlines the study protocol and baseline characteristics of the study population. The main aims of this study are as follows: (1) to determine the effect of a 2-year digital, multi-domain AD risk reduction intervention on the overall risk of AD in adults at risk of developing the disease; (2) to determine the effect of digital interventions on the rate of cognitive decline; and (3) to determine the effect of digital interventions on the changes in general health outcomes.

## Methods

### Study Design and Participants

This is a single-site, 2-year randomized controlled trial (RCT). The participants include men and women aged from 45 to 75 years who have risk factors for dementia. Each participant has been informed of the purpose of the intervention and has agreed to be assigned randomly into 1 of the 2 study groups (HC or HE).

To be included in the study, the participants must have at least 2 positive risk factors for AD as determined by the Australian National University-Alzheimer Disease Risk Index (ANU-ADRI) [11] and not more than 1 protective factor (eg, high level of physical activity). Textbox 1 shows the complete list of the inclusion and exclusion criteria. The participants will complete 4 study visits over 2 years: at baseline and 4, 12, and 24 months.

**Inclusion and exclusion criteria.**

**Inclusion criteria**

- Age: 45-75 years
- BMI: 18.5-39.9 kg/m2
- Fluent in English (written and spoken)
- At least 2 of the following risk factors for Alzheimer disease (AD) from Australian National University-Alzheimer Disease Risk Index (ANU-ADRI):
  High school education or less
  Overweight or class I or class II obese (BMI 25-39.9 kg/m2)
  History of diabetes, hypertension, high cholesterol, smoking, or traumatic brain injury
- At most, 1 of the following protective factors for AD from ANU-ADRI:
  High level of physical activity (as defined by the high International Physical Activity Questionnaire category)
  High fish consumption (as defined by consumption of fish or seafood that is not fried, for >5-6 times per week)
  High level of cognitive engagement (as defined by engaging in at least 6 of the following activities several times a week: reading a book, newspaper, or magazine; playing brain games; playing games; writing letters or emails; participating in web-based social network activities; attending a concert, play, or musical; or visiting a library)
- Ability to send and receive SMS text messages
- Own a smartphone with a reliable internet connection and willing to use email
- Ability to participate in light to moderate physical activity
- Willing to be randomized

**Exclusion criteria**

- Physician diagnosis of the following:
  Mental health condition (eg, eating disorder, alcohol and substance use, and schizophrenia)
  Neurologic conditions (eg, epilepsy, stroke, multiple sclerosis, Parkinson disease, brain tumor, or severe traumatic brain injury)
  Dementia, probable dementia, or mild cognitive impairment
  Other significant health condition (eg, congestive heart failure, chronic obstructive pulmonary disease, coronary artery disease, renal failure, chronic kidney disease, and pulmonary hypertension)
- Recent cardiovascular event or recent treatment for cancer (within the last year), on dialysis, or on active organ transplant list
- Visual problems that prevent viewing screen at a normal distance (eg, legal blindness, detached retina, and occlusive cataracts)
- History of learning disability
- Currently participating in a formal cognitive training coaching program or other lifestyle change program (eg, diabetes prevention program)
- Currently pregnant or planning to become pregnant in the next 2 years
- Not meeting all the inclusion criteria

During the first visit, after eligibility was confirmed and informed consent was obtained, the participants completed a baseline survey. The survey contained questions about demographics (age, race or ethnicity, and education level), contact information, sleep, stress, anxiety, depression, general well-being, and dementia risk. Then, the participants completed a set of cognitive assessments, including the Repeatable Battery for the Assessment of Neuropsychological Status (RBANS) [19] and the Neurotrack digital assessments. Next, the participants completed a series of physical evaluations, including the Short Physical Performance Battery, 6-minute walk test (6MWT), dual-task, hand grip, and sit-to-stand lower-body power assessment. Finally, biometric data were collected, including body composition: BMI, body fat using dual-energy x-ray absorptiometry, and resting heart rate; blood pressure; fasting blood glucose; lipid panel—total cholesterol, high-density lipoprotein (HDL), low-density lipoprotein (LDL), and triglycerides; brain-derived neurotrophic factor; interleukin-6; high-sensitivity C-reactive protein; and APOE status. Reassessment will occur after 4, 12, and 24 months and will include the collection of most but not all of the data collected at baseline (Table 1).

**Table 1:** Summary of measures collected at each study visit.

Category and measurement			Baseline		4 months		12 months		24 months
Informed consent			Yes^a^		No^b^		No		No
**Demographics**									
	Age (years)	Yes		No		No		No	
	Race	Yes		No		No		No	
	Education level	Yes		No		No		No	
	Marital status	Yes		No		No		No	
Resting blood pressure (heart rate)			Yes		Yes		Yes		Yes
Supplement and medication list			Yes		Yes		Yes		Yes
**Body composition**									
	Height and weight; BMI	Yes		Yes		Yes		Yes	
	Body fat (DXA^c^)	Yes		Yes		Yes		Yes	
**Bloodwork**									
	Lipids (LDL^d^, HDL^e^, TC^f^, and triglycerides)	Yes		Yes		Yes		Yes	
	Glucose	Yes		Yes		Yes		Yes	
	BDNF^g^	Yes		Yes		Yes		Yes	
	hs-CRP^h^	Yes		Yes		Yes		Yes	
	IL-6^i^	Yes		Yes		Yes		Yes	
**ANU-ADRI^j^**									
	Smoking and drinking habits	Yes		Yes		Yes		Yes	
	Exercise and dietary patterns	Yes		Yes		Yes		Yes	
	History of diabetes, depression, high cholesterol, and TBI^k^	Yes		Yes		Yes		Yes	
	Social engagement and cognitive activity	Yes		Yes		Yes		Yes	
	Fish intake	Yes		Yes		Yes		Yes	
	Pesticide exposure	Yes		Yes		Yes		Yes	
**RBANS^l^** **(total and subscales)**									
	Immediate memory	Yes		Yes		Yes		Yes	
	Delayed memory	Yes		Yes		Yes		Yes	
	Visuospatial and constructional abilities	Yes		Yes		Yes		Yes	
	Language	Yes		Yes		Yes		Yes	
	Attention	Yes		Yes		Yes		Yes	
**Neurotrack digital assessments**									
	Image pairs	Yes		Yes		Yes		Yes	
	Symbol match	Yes		Yes		Yes		Yes	
	Item price	Yes		Yes		Yes		Yes	
	Arrow match	Yes		Yes		Yes		Yes	
	Light reaction	Yes		Yes		Yes		Yes	
	Path points	Yes		Yes		Yes		Yes	
	Digital choice anxiety survey	Yes		Yes		Yes		Yes	
**Functional fitness measures**									
	SPPB^m^	Yes		Yes		Yes		Yes	
	6MWT^n^	Yes		Yes		Yes		Yes	
	Dual-task	Yes		Yes		Yes		Yes	
	Hand grip	Yes		Yes		Yes		Yes	
	TENDO sit-to-stand	Yes		Yes		Yes		Yes	
**Behavioral, quality of life, and health care use questions**									
	Everyday Cognition (E-Cog-12^o^)	Yes		Yes		Yes		Yes	
	Sleep (PSQI^p^)	Yes		Yes		Yes		Yes	
	Stress (PSS^q^)	Yes		Yes		Yes		Yes	
	Well-being (SF-12^r^)	Yes		Yes		Yes		Yes	
	Loneliness (UCLA^s^)	Yes		Yes		Yes		Yes	
	Depression (PHQ-9^t^)	Yes		Yes		Yes		Yes	
	Anxiety (GAD-7^u^)	Yes		Yes		Yes		Yes	
	Health care use (UCSD^v^)	Yes		Yes		Yes		Yes	
APOE^w^ status			Yes		No		No		No

^a^Will be measured.

^b^Will not be measured.

^c^DXA: dual-energy x-ray absorptiometry.

^d^LDL: low-density lipoprotein.

^e^HDL: high-density lipoprotein.

^f^TC: total cholesterol.

^g^BDNF: brain-derived neurotropic factor.

^h^hs-CRP: high-sensitivity C-reactive protein.

^i^IL-6: interleukin 6.

^j^ANU-ADRI: Australian National University-Alzheimer Disease Risk Index.

^k^TBI: traumatic brain injury.

^l^RBANS: Repeatable Battery for the Assessment of Neuropsychological Status.

^m^SPPB: Short Physical Performance Battery.

^n^6MWT: 6-minute walk test.

^o^E-Cog-12: 12-item Everyday Cognition Scale.

^p^PSQI: Pittsburgh Sleep Quality Index.

^q^PSS: Perceived Stress Scale.

^r^SF-12: 12-Item Short Form Health Survey.

^s^UCLA: University of California, Los Angeles, 3-item Loneliness Scale.

^t^PHQ-9: 9-item Patient Health Questionnaire.

^u^GAD-7: General Anxiety Disorder 7-item scale.

^v^UCSD: University of California at San Diego.

^w^APOE: apolipoprotein E.

### Cognitive Testing

#### RBANS Test Battery

The cognitive function of the participants will be assessed using RBANS [19] at all the 4 time points. Details of this test battery are published elsewhere [19,20]. In brief, memory (immediate and delayed), visuospatial and construction, attention, and language will be tested using a digital (iPad) platform. Reliability (*r*=.81) and validity (*r*=.59) of the RBANS test battery were previously demonstrated with global cognition scores when tested with community-dwelling older adults [21].

#### Neurotrack Test Battery

Neurotrack Technologies has developed a digital battery of cognitive assessments measuring attention, associative learning, memory, inhibition, executive function, and processing speed. The Neurotrack assessments were found to be valid (*r*=.57), reliable (*r*=.73), and able to discriminate between older adults who are cognitively normal and cognitively impaired [22,23].

#### Item Price Test

Associative learning and memory will be assessed using the Item Price test. This assessment will consist of a familiarization phase in which the items (eg, various fruits and vegetables) will be presented along with their associated prices. Immediately after the familiarization phase, the participants will be presented with the items and a corresponding price. The participants will be instructed to select *yes* or *no* depending on whether the item price matches the previously viewed amounts. In total, 60 trials will be presented, including 24 targets (images previously paired), 24 foils (images previously present but not paired), and 12 shams (images not presented). Scores will be reported as the accuracy of identifying the correct item based on its price.

#### Image Pairs Test

Image pairs is an eye-tracking task that measures visual recognition memory and learning [22,23]. The participants will be presented with 110 images, categorized into 4 phases. Phase 1 will be a familiarization phase consisting of 20 images. During phase 2, the participants will be presented with 2 images—1 novel and 1 previously viewed in phase 1. During this phase, the participants will be instructed to focus their gaze on the novel image. Phase 3 will consist of a learning phase in which the participants will be presented with 2 images and will be asked to remember their association with each other as pairs. Phase 4 will consist of 50 trials with 20 targets from phase 3, 20 foils, and 10 sham trials. During this phase, the participants will be instructed to select *yes* or *no* to identify if the presented images were previously viewed together as a pair. This test will measure the participant’s ability to learn and identify image pairs. Scores for phase 2 will be reported as the percentage of time spent on gazing at the novel image, and for phase 4, it will be reported as accuracy.

#### Symbol Match Test

Symbol match is a processing speed and executive functioning task that uses a paired verification or rejection paradigm (forced choice). The participants will be instructed to determine whether 2 symbols are equal or unequal using a legend with 9 number or symbol pairs. The participants will be allotted 2 minutes to complete as many trials as possible. Scores will be determined by the number of correct trials minus the number of incorrect trials.

#### Arrow Match Test

Arrow Match test is a measure of attention and processing speed. The participants will be shown 5 arrows in the middle of the screen and will be instructed to identify the direction of the middle arrow. The arrow may point in either the same direction or the opposite direction from the other arrows. The participants will be presented with 32 trials, and the scores will be reported as the number of correct responses relative to the time elapsed during all the trials.

#### Path Points Test

Executive function will be assessed using the Path Points test. Similar to the paper-pencil Trail Making Test Part B [24], the Path Points test is a digital version in which the participants will be instructed to connect a series of alternating numbers and letters from 1-A to 7-G. Scores will be reported as the duration required to complete the 14 responses. Only correct responses will be considered for scoring.

#### Light Reaction Test

Reaction time and inhibition will be assessed using the Light Reaction test. The participants will be presented with either a positive (green light) or a negative stimulus (red light). The participants will be instructed to press a button if the positive stimulus appears and to refrain from pressing the button if the negative stimulus appears. The average response time for reacting to the positive stimulus (green light) will be recorded.

### Physical Function Assessments

Short Physical Performance Battery consists of 3 assessments: standing balance, usual walk time, and chair stand performance. The standing balance score will be compiled from 3 balance tests: standing with feet together, standing in a semitandem position, and standing in a full tandem position. If the participant could stand with their feet together and in the semitandem position for 10 seconds, 1 point will be given for each condition. If the participant stands for 10 seconds in the full tandem position, 2 points will be given, 1 point for 3-9.99 seconds, and 0 points if they do not hold the position for at least 3 seconds. For the assessment of usual walk time, the participants will be instructed to walk at their usual pace for 4 m. Scores will range from 0 to 4 based on the time to completion [25,26]. The chair stand test will be performed in a standard straight-back chair (seat height=0.43 m). The participants will be instructed to sit with both feet on the floor with arms crossed over their chest. The time to complete 5 chair stands will be recorded, with scores ranging from 0 to 4. Each component (balance, walking time, and chair stand) will be summed to generate a composite score; scores <10 indicate physical dysfunction.

Cardiovascular endurance will be assessed using the 6MWT [27,28]. The 6MWT will be performed in a well-lit hallway with cones separated by 25 m. The participants will be instructed to walk as quickly as possible for 6 minutes. The distance covered within the 6 minutes will be recorded. The 6MWT is a valid and reliable estimate of aerobic fitness [28].

Hand grip strength will be assessed using a Takei hand grip dynamometer (Takei Scientific Instruments Co, Ltd). The participants will be properly fit and instructed to squeeze maximally for at least 3 seconds. Verbal encouragement will be provided. Three trials will be completed on each hand with a 60-second rest between trials. Hand grip strength is positively correlated with overall muscle strength and physical mobility [29-31].

Gait speed will be determined using 2 trials: habitual and fast. For the habitual trial, the participants will be instructed to walk 20 m at their habitual or usual walking speed. Immediately after the habitual speed trial, the participants will be instructed to walk as quickly and as safely as possible without running. Two trials will be completed for both conditions and only the middle 10 m gait speed distance will be recorded and used for all the analyses. Gait speed was previously found to be a valid and reliable measure of physical mobility [32] and cognition [33].

Immediately after the gait speed trials, the participants will be instructed to repeat both habitual and fast trials while completing a serial subtraction cognitive task. The participants will be given a randomly generated 3-digit number ranging from 100 to 999 and will be instructed to begin walking immediately upon receiving their number. The participants will walk the entire distance while subtracting 3 from their assigned number aloud. Both, time to cover the 10 m distance and correct and incorrect numbers will be recorded [34-37].

Lower extremity muscular power will be assessed using a power chair stand [38-40]. The participants will be instructed to sit on a chair of standard height (0.43 m) with both feet flat on the floor and arms crossed over their chest. Power (peak and average) and velocity (peak and average) will be measured using TENDO (Tendo Sport). Five trials will be completed, and the average scores will be used for all analyses.

### Surveys

#### ANU-ADRI Tool

ANU-ADRI is an evidence-based 79-item risk assessment tool designed to predict the risk of future AD development. ANU-ADRI collects information on education, BMI, cholesterol, diabetes, history of traumatic brain injury, depression, physical activity, cognitive engagement, social network, fish intake, alcohol consumption, smoking, and pesticide exposure [11]. This valid and reliable tool will be used as the primary inclusion tool and a primary outcome variable in this study. Details of the ANU-ADRI scoring procedures are published elsewhere [11]. Briefly, scores for each subsection will be tallied and a composite risk score will be used for all the analyses. A change of 2 points in the ANU-ADRI score is considered clinically meaningful [41].

#### Anxiety

Anxiety will be assessed using the General Anxiety Disorder 7-item scale [42]. It is a valid and reliable measure of anxiety among older adults [43]. Scores range from 0 to 21, with higher scores indicating higher anxiety severity [42].

#### Loneliness

The University of California, Los Angeles, 3-item Loneliness Scale [44] will be used to determine the degree of loneliness among the study population. Scores range from 3 to 9, with higher scores being associated with greater levels of loneliness. Scores above 5 indicate loneliness.

#### Health-Related Quality of Life

Health-related quality of life will be measured using the 12-Item Short Form Health Survey, which is a valid and reliable measure of health-related quality of life in many study populations [45]. It is composed of 2 subscales: physical health and mental health. Scores range from 12 to 47, with higher scores indicating higher self-reported quality of life.

#### Perceived Stress

Perceived stress will be assessed using the Perceived Stress Scale [46]. Scores range from 0 to 40, with lower scores indicating lower perceived stress. Scores ranging from 0 to 13 indicate low stress, 14 to 26 indicate moderate stress, and >26 indicate high stress.

#### Physical Activity

Physical activity will be assessed using the International Physical Activity Questionnaire, which is the physical activity component of the ANU-ADRI. This survey is a valid and reliable self-report tool for quantifying moderate, vigorous, and sedentary behaviors [47]. The International Physical Activity Questionnaire specifically asks about the participant’s physical activity performed within the previous 7 days.

#### Health Care Use

The health care use form of the University of California at San Diego will be used to quantify how frequently the participants have visited their physician or used any form of health care within the previous 3 months. Higher values indicate more health care use during the time frame [48].

#### Sleep

Sleep quality will be assessed using the Pittsburgh Sleep Quality Index. This 9-item assessment produces a score ranging from 0 to 27, with higher scores indicating poorer sleep. Individuals scoring ≥5 are deemed poor sleepers [49,50].

#### Depression

Depression will be assessed using 2 surveys. The Patient Health Questionnaire is a 9-item questionnaire with scores ranging from 0 to 27. Higher scores indicate higher levels of depression [51]. The Center for Epidemiological Studies-Depression is a 20-item depression scale. Higher values indicate higher levels of depression, with a score ≥16 indicating individuals at risk for clinical depression. The Center for Epidemiological Studies-Depression is a part of the ANU-ADRI [11].

#### Everyday Cognition

The 12-item Everyday Cognition scale is a brief questionnaire designed to detect cognitive and functional decline. This scale has been shown to correlate with functional measures and neuropsychological scores in people with normal cognitive function, mild cognitive impairment (MCI), and AD [52].

#### Blood Biomarkers

Blood sample will be collected at each of the 4 study visits. High-sensitivity C-reactive protein, interleukin-6, and brain-derived neurotrophic factor will be analyzed by a third-party laboratory. Cholesterol (total, HDL, and LDL), triglycerides, and blood glucose levels will be analyzed in whole blood using a Cholestech LDX system (Abbott Laboratories). Whole blood will be collected in a 40 µL capillary tube, immediately transferred into a Cholestech cartridge, and analyzed. Cholestech LDX values are valid and reliable for the assessment of triglycerides, LDL, HDL, and total cholesterol [53]. APOE status will be analyzed at baseline only.

### Study Arms

#### Randomization

Before the recruitment of participants into the intervention, all the study ID numbers were randomly preassigned. A member of the research team assigned each study ID to a number generated by a random numbers table. As participants (who met all the inclusion criteria and none of the exclusion criteria and signed the informed consent form) were enrolled in the study, they were assigned a study ID number.

#### HC Intervention

The participants randomly allocated into the HC arm of the study will be assigned a personal health coach to work with for the duration of the study. HC will take place remotely through videoconferences and asynchronous chat messages and focus on helping the participants improve their brain health by working on the following lifestyle domains: nutrition, physical activity, sleep, stress, social engagement, and cognitive activity. HC will communicate with the participants about each of these modifiable risk factors and then tailor their recommendations and communications to the specific lifestyle areas needed for each participant. HC participants will also be provided access to a cognitive health app (Citruslabs), through which they can access cognitive training activities, workout routines, and recipes. HC is different from traditional interventions used in RCTs. Instead of testing a uniform program to fit all the participants, the HC recommendations and communications will be tailored by the coach to fit each participant’s unique needs.

The HC will actively reach out to participants 1-2 times per week through asynchronous messages and will provide articles on various lifestyle modifications, such as nutrition information and physical activity, based on the focus for each participant. In addition, the participants will have unlimited access to their coach to ask questions or obtain the coach’s recommendations. Meetings with the coach will be scheduled on a monthly basis through videoconference or phone to assess the progress toward the goals, discuss any barriers they have encountered, and strategize personalized ways to attain the goals. The lifestyle domains will be chosen based on a combination of the participant’s preferences and the coach’s recommendations. For example, if the HC identifies nutrition as the primary source of need for a participant but the participant is not ready to work on that area, the HC will make alternative recommendations to meet the participant where they feel ready to make a change.

The HC intervention is designed to support participants through the recommended lifestyle changes to improve brain health and reduce dementia risk. The topics that will be discussed during the initial intake session include (1) description of the HC process, (2) description of lifestyle domains and how they are related to brain health, (3) gathering information from the participant on which lifestyle domains they seek and are willing to improve, (4) assessing motivation and readiness for change, and (5) assisting the participant for creating a vision for their future, including appropriate goal-setting. Although the monthly follow-up coaching sessions can differ between participants, they will typically include (1) reassessing goals and making appropriate modifications to meet personal health goals and (2) supporting the participant in overcoming the challenges they meet throughout their personal journey.

#### HE Control

Participants randomly assigned to the HE control group will receive biweekly emails including information on how to change their lifestyle to improve their brain health. To independently evaluate the efficacy of the intervention, the same lifestyle domains will be covered in both arms of the study. These lifestyle domains include physical activity, dietary habits, sleep, stress, social engagement, and cognitive activity. Throughout the duration of the intervention, the participants will only have access to the study staff during their on-site testing sessions. After the intervention, the number of articles opened by the participant will be tallied to determine engagement from the control group.

### Participant Recruitment

Participants were recruited in northwest Arkansas using local radio, email, social media advertisements, and word of mouth. The participants who met all the inclusion criteria and none of the exclusion criteria were randomly assigned to one of the groups before any assessments were completed.

The goal sample size for this study is approximately 200 participants. This sample size is considered sufficient to achieve random assortment, which was validated after examining the baseline characteristics of both groups. Cross-sectional, between-group differences in the baseline ANU-ADRI scores will be assessed using analysis of variance (ANOVA). Longitudinal studies following cognitively intact older adults have documented a 0.8 RBANS standard score change (SD 13.8) over a 12-month period [54]. Owing to the younger and wider age range to be recruited, we conservatively estimated a 1.75 standard score change with a comparable SD 10 among the control participants. In a 12-month pilot study of a similar intervention, a 5.8 (SD 7.4) standard score point increase on RBANS (ie, improvement) was found [55]. Assuming no additional improvement owing to a longer intervention, a sample size of 100 participants per group provides 80% power to detect a difference of 3.3 points between the intervention groups on the RBANS total index score (Cohen *d*=0.33) with an α of .05 and accounting for up to 20% attrition.

### Statistical Analyses

Using the results of previous web-based intervention studies designed to reduce AD risk in an RCT design [11,56], a 2-point difference in the ANU-ADRI total score between the groups is expected over time [41]. A longitudinal observational study among adults demonstrated that a 1-point increase in ANU-ADRI score is associated with an 8% increase in the risk for developing MCI or dementia, mediated by brain volume, over the following 12 years [57]. A 2.5-point within-group change was found to be statistically significant in a web intervention [11]. Owing to the population health effects that are possible with a scalable remote intervention, these metrics are clinically and statistically meaningful at the individual and group levels.

Exploratory analyses will be performed on an intent-to-treat basis. One-way ANOVA will be used to compare the groups on functional fitness; biomarker measures; health-related variables; and digital, cognitive assessments at baseline. In addition, sex differences in baseline characteristics and change scores will be evaluated using a 2×2 (group and sex) ANOVA. Repeated measures ANOVA will be used to determine the differences over time between the groups. APOE status and the presence of homozygous E4 alleles will be used as a dichotomous variable and as a covariate for change in results after the intervention.

### Ethics Approval Statement

The study was approved by the University of Arkansas Institutional Review Board. All participants signed an approved consent form in accordance with the ethical standards of Helsinki.

## Results

**Figure 1:**
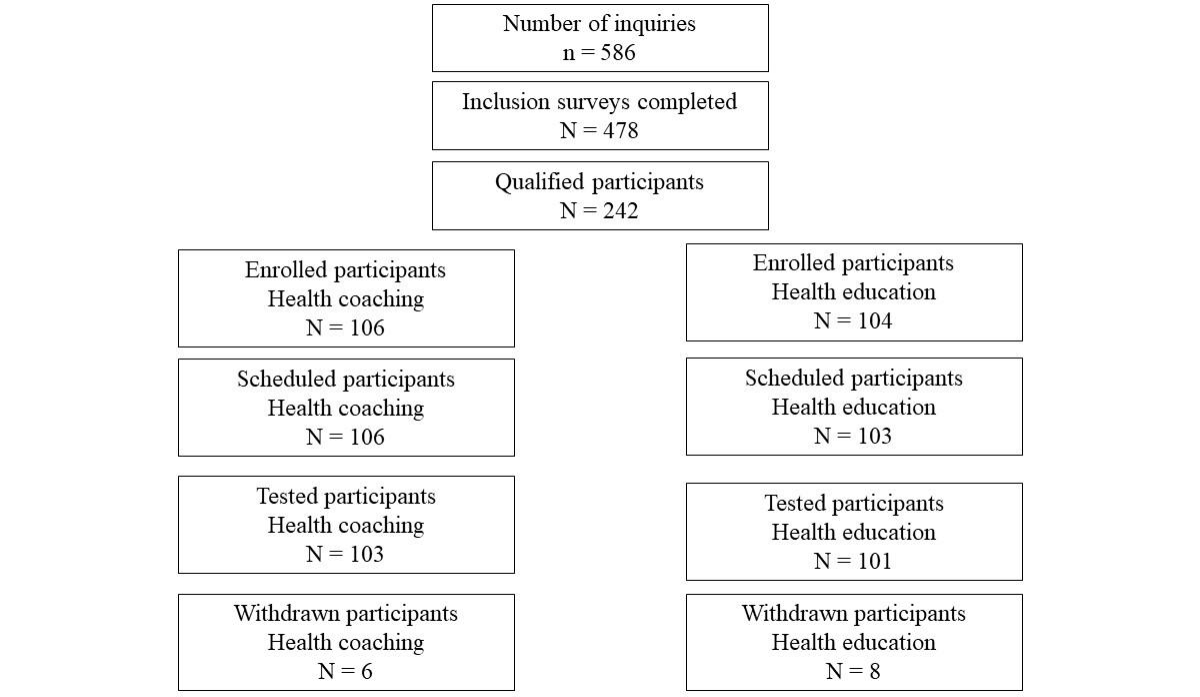
Participant screening and enrollment funnel.

The enrollment funnel for the DCMARVel trial is shown in Figure 1. Enrollment took place between January 2021 and June 2021. The baseline characteristics of the study population are presented in Table 2. A total of 204 adults (n=152, 74.5% women and n=52, 25.5% men) have completed the baseline testing. The participants are aged 45-75 years (mean 61.9, SD 8.3 years).

**Table 2:** Baseline characteristics (N=204).

Variables	Health education (n=101)	Health coaching (n=103)	Total (N=204)	*P* value^a^
Age (years), mean (SD)	61.4 (8.9)	62.4 (7.6)	61.9 (8.3)	.38
Sex (female), n (%)	77 (76.2)	75 (72.8)	152 (74.5)	—^b^
Ethnicity (White), n (%)	98 (97)	100 (97.1)	198 (97.1)	—
ANU-ADRI^c^ total, mean (SD)	−1.61 (7.34)	−1.75 (7.32)	−1.68 (7.31)	.90
ANU-ADRI risk, mean (SD)	8.97 (5.91)	8.41 (5.57)	8.68 (5.73)	.48
ANU-ADRI protective, mean (SD)	−10.38 (4.49)	−10.15 (4.60)	−10.26 (4.54)	.72

^a^Obtained from 2-tailed *t* test.

^b^No statistical analysis was performed.

^c^ANU-ADRI: Australian National University-Alzheimer Disease Risk Index.

## Discussion

### Principal Findings

To our knowledge, this study is the first large-scale 2-year RCT to examine the effect of a digital multi-domain lifestyle intervention on reducing AD risk among a population that includes adults as young as 45 years. Improving the modifiable risk factors for AD has the greatest potential to impact disease development when implemented early and in a targeted manner and effective treatments hinge upon early identification and appropriate lifestyle modification. Although several single-domain interventions have been implemented with little success, multi-domain interventions have demonstrated greater levels of improvement [8,15,16]. However, many of these interventions have focused on cognitive change among older adults at risk for dementia [8,16]. In addition, the interventions have largely been designed for face-to-face implementation; therefore, greatly reducing the scalability and public health impact of the programs. Thus, this study will focus on impacting multiple lifestyle domains linked to AD risk in a younger population than that studied previously [8,9,17,18].

The HC intervention will address 6 lifestyle domains that have been linked to the development of dementia later in life: diet, exercise, sleep, stress, social engagement, and cognitive activity. In addition to receiving HE material similar to the control group, participants in the intervention arm will work with a health coach to develop and implement changes to the lifestyle areas that they are ready to work on. The tailored nature of the program is designed to maximize the behavior change outcomes.

Dietary habits are an important modifiable risk factor for dementia. The Mediterranean-DASH intervention for neurodegenerative delay (MIND) diet is recommended to participants based on its positive effects on brain health [9,58]. Even moderate adherence to the MIND diet has been linked to better brain health and reduced dementia risk in the long term. In the studies by Morris et al [9,58], participants in the top tertile of the MIND diet scores showed a 53% reduction in the rate of developing AD compared with participants in the lowest tertile and participants in the middle tertile also showed a statistically significant 35% reduction in AD rate compared with those in the first tertile. The diet is also easy to follow because it emphasizes eating more of the recommended foods, such as berries and leafy greens, and less of the unhealthy foods, such as butter and sweets, rather than requiring a strict eating plan. This type of dietary pattern will allow the HC to make substitutions, modifications, and adjustments in the recommendations based on dietary needs and preferences.

Habitual physical inactivity is associated with many chronic health conditions such as type 2 diabetes, cardiovascular disease, and hypertension [59]. More recently, research has suggested that higher cardiorespiratory fitness and physical activity participation have a positive association with cognitive performance [59,60]. Acute exercise has been associated with improved performance in many cognitive paradigms, but the greatest effect has been noted on executive function and reaction time and smaller effects have been noted on memory and processing speed [60,61]. The physical activity recommendations made by the HC will be determined by the participant’s current level of exercise and their readiness to do more.

Sleep is an important aspect of both brain health and overall health. Aging is often associated with changes in sleep patterns [62]. The causes of these changes are not well understood, but have been linked to reductions in neurocognitive function [63]. In addition, adults with dementia or AD often experience significant disturbances in sleep quality and sleep patterns [62,63]. Although it is well-recognized that sleep disturbances are common among adults with dementia and AD, identification of sleep disturbances earlier in life may be predictive of future cognitive decline [62]. The HC will work with participants to identify any existing sleep issues and implement strategies to improve the quality and quantity of sleep.

Stress is a multifactorial response to both internal and external stimuli. However, exposure to chronic stress can result in major illnesses and ailments [64,65]. Many studies have linked the exposure to chronic stress with cardiovascular disease [64] and cognitive dysfunction [66]. The specific mechanisms underlying this link have not yet been fully elucidated. However, a potential mechanism includes stress hormones that decrease the glutamate receptor function in the prefrontal cortex [67]. This is important because the prefrontal cortex is responsible for many aspects of executive function, including working memory and inhibition [68]. The participants will work with the HC on personalized strategies to reduce stress based on their individual circumstances and needs.

Loneliness has recently been identified as a positive predictor of dementia among older adults. After a 3-year follow-up, older adults with self-reported feelings of loneliness and living alone showed an approximately 2.5-fold increase in the risk of dementia [69]. A recent meta-analysis supports these results by suggesting that the relative risk of dementia is 25% higher among older adults with feelings of loneliness when compared with those with a greater social network [70]. The HC will work with the participants to assess their level of social engagement or loneliness and make changes as needed.

Low cognitive engagement has previously been linked with increased risk of dementia [71,72]. This particular risk factor has gained significant attention in recent years because it is one of the easiest risk factors to be modified. Cognitive engagement or brain training research has increased significantly over the past few years, but the results have been inconclusive. Bahar-Fuchs et al [73,74] examined changes in global cognition after 8 weeks of cognitive training and found improvements in cognitive function that persisted during a 6-month follow-up period. Edwards et al [75] found that training cognitive processing speed lowered the risk of developing dementia by 29%, whereas memory training and cognitive reasoning reduced dementia risk by 21% [75]. It is likely that sustained participation in cognitively challenging activities, such as learning an instrument or doing crossword puzzles, has a positive impact on brain health in the long term [76-78]. The health coach will work with the participants to identify activities that are of interest to them and encourage regular practice.

### Conclusions

The results of this study can produce a novel and highly scalable intervention strategy to reduce the risk of cognitive decline before MCI or AD diagnoses occur. The primary outcome in the DCMARVel trial is AD risk reduction as determined by ANU-ADRI [11]. With the wide range of secondary outcomes (eg, physical, blood biomarkers, and psychosocial), the relative contribution to AD risk reduction can be estimated in addition to changes over time. As the HC program is designed to address multiple factors contributing to vascular disease (eg, physical activity and dietary habits), the intervention is expected to improve cognitive outcomes over time by significantly reducing AD risk using a digital and scalable format.

## Abbreviations

6MWT: 6-minute walk test
AD: Alzheimer disease
ANOVA: analysis of variance
ANU-ADRI: Australian National University-Alzheimer Disease Risk Index
APOE: apolipoprotein E
DCMARVel: Digital, Cognitive, Multi-domain Alzheimer Risk Velocity
HC: health coaching
HDL: high-density lipoprotein
HE: health education
LDL: low-density lipoprotein
MCI: mild cognitive impairment
MIND: Mediterranean-DASH intervention for neurodegenerative delay
RBANS: Repeatable Battery for the Assessment of Neuropsychological Status
RCT: randomized controlled trial
